# Amalgam Tattoo Mimicking Mucosal Melanoma: A Diagnostic Dilemma Revisited

**DOI:** 10.1155/2013/787294

**Published:** 2013-03-06

**Authors:** K. Lundin, G. Schmidt, C. Bonde

**Affiliations:** Department of Plastic Surgery, Breast Surgery and Burns Treatment, Rigshospitalet-Copenhagen University Hospital, 2100 Copenhagen OE, Denmark

## Abstract

Mucosal melanoma of the oral cavity is a rare but highly aggressive neoplasm. However, the clinicians need to be aware of the other and more frequent etiologies of intraoral pigmentation, such as amalgam tattoos. As amalgam has been extensively used for dental restorations and can cause pigmentations in the oral mucosa, this is a differential diagnosis not to be forgotten. We describe the characteristics of these two phenomena and present a case vignette illustrating the differential diagnostic issues. Other causes of intraoral pigmentation are summarized.

## 1. Introduction

Mucosal melanoma of the oral cavity is a highly aggressive neoplasm. The incidence and clinical course have been described in several studies despite its rarity [1–4]. However, the other etiologies of intraoral pigmentation need to be considered by the clinician. We here describe the characteristics of melanoma of the oral mucosa and the so-called amalgam tattoos. A case vignette of amalgam tattoo mimicking the more dire diagnosis of mucosal melanoma is presented, and other causes of intraoral pigmentation are summarized.

## 2. Case Vignette

A 51-year-old woman was referred to the Department of Plastic Surgery on suspicion of mucosal melanoma. She was a heavy smoker but otherwise healthy, with no prior family or personal history of melanoma.

The patient's dentist had noticed two dark discolorations in the gingiva from which the patient had no symptoms, though she suffered greatly from pain localised in the right side of the maxilla and mandible. The pain was thought to originate from extensive prosthetic dental treatment during the last six months. 

**Figure 1:**
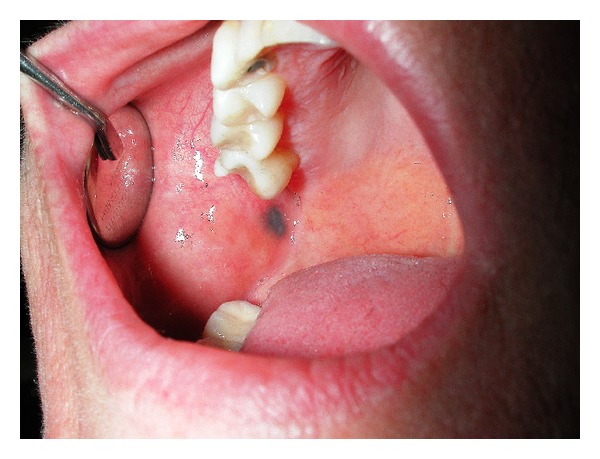
Clinical photograph of the 3 × 5 mm pigmented lesion of the left buccal mucosa mimicking mucosal melanoma.

**Figure 2:**
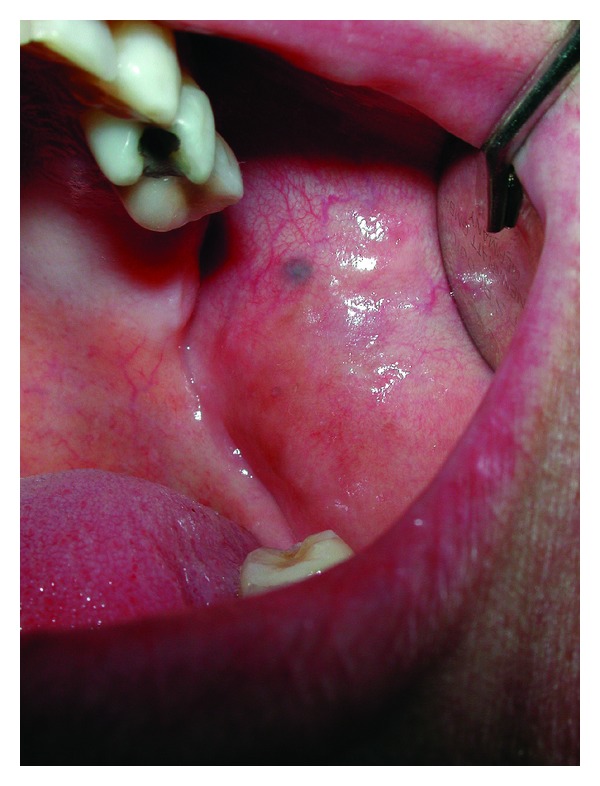
Clinical photographs of the 2 × 2 mm pigmented lesion of the right buccal mucosa. Histopathological examination was consistent with amalgam tattoo in both lesions (see Figure 1).

The bluish-grey discolorations of the mucosa were localized in the buccal mucosa opposite the third upper molar on the right side and the first upper molar on the left side as seen in clinical photographs (Figures 1 and 2). No other suspect lesions or any enlarged lymph nodes were found, and the patient was treated with narrow excision of the elements under local anaesthesia. 

Histopathological examination of the specimens showed brownish-black pigment along the collagenous fibres and in the vascular sheaths. No melanocytes or naevus cells were found, and there was no positive reaction in melanin stains. Both lesions were found to be consistent with amalgam tattoos. No other macules were identified in the oral mucosa, and the patient needed no further treatment or follow-up.

## 3. Discussion

Mucosal melanoma is very rare and reports are scarce, but it is considered one of the most aggressive malignancies known [1–7]. The recorded incidence is up to 1 or 2% of all melanomas [1–4, 7]; it is seen typically in the 4–7 decade and with no certain difference between sexes [1, 2, 4–6]. It is more frequent in African and Asian populations compared to Caucasians [1, 3, 5, 7, 8].

Clinical signs of mucosal melanoma of the oral cavity are usually dark brown, black, or bluish-greyish plaques with irregular pigmentation and an asymmetrical, irregular border [5, 6]. Swelling, ulceration, bleeding, pain/discomfort, and ill-fitting dentures are also common [2, 3, 5, 6]. Oral mucosal melanomas are typically of lentiginous or superficial type but may also be nodular [3, 9]. Most are localized to the maxillary mucosa and palate [2–6, 8]. Five to fifteen percent are reported to be amelanotic, though one study described up to two-thirds [2, 3, 5, 6, 8]. Mucosal melanomas are often preceded by a pigmented premalignant lesion, but due to the location, they tend to be diagnosed late and metastatic disease is not uncommon at the time of diagnosis [3, 5, 8]. Prognosis is uniformly described as poor and has been cited as below 15% five-year survival for oral mucosal melanoma [1]. 

However, studies are small and cannot provide certain stage-specific survival rates due to the rarity of the disease. Treatment is to a certain degree extrapolated from the regimens used for cutaneous melanoma [2, 9]. First choice treatment is wide radical excision. Though sentinel lymph node biopsy is state of the art for cutaneous melanoma, its role in mucosal melanomas remains uncertain and is not part of the recent guidelines from the National Comprehensive Cancer Network [1, 9–12]. Treatment of the clinically node-negative neck also remains controversial. Radiation has been used as adjuvant postoperative therapy and is also used for palliation [1, 12]. For metastatic disease, systemic therapy is suggested [1, 3, 4, 12]. 

By contrast, amalgam tattoos (formerly called localized argyria) is one of the most frequent causes of exogenous pigmentation in the oral mucosa and is not uncommon to present as two or more lesions [5, 13–15], as seen in the present case. Amalgam consists of an alloy of liquid mercury with varying amounts of silver, tin, copper, and zinc. Amalgam tattoos are usually caused by amalgam splinters inadvertently implanted into the mucosa during dental restorations [5, 7, 13, 15] but may also be caused by diffusion through the teeth [15]. As amalgam has been the most commonly used material for dental fillings until the 1980s, there is still a large amount of prosthetic work being done on existing amalgam fillings. Thus the prevalence of amalgam tattoos remains high as the general population continues to have existing amalgam fillings replaced by the newer composite fillings. Depending on the depth in the tissue, the deposits of amalgam in the mucosa may be visible and present macroscopically as a localised pigmented area. Clinically, this phenomenon presents as grey, blue, or black, nonblanching macules in the oral mucosa [5, 13, 15]. Their appearance can be difficult to discern from other pigmented elements of the oral mucosa including mucosal melanoma.

Histologically, deposits of amalgam are seen as granules along blood vessels and collagen fibres or as solid fragments in the tissue. There may be an associated foreign-body reaction [7, 13]. 

Amalgam tattoos are harmless and asymptomatic. They can be safely diagnosed by the finding of radio-opaque granules on X-ray, but large particles of amalgam must be present in order for this method to be useful [5–7, 13]. Many endogenous and exogenous conditions can cause pigmentation of the oral mucosa; see Table 1. Pigmented lesions of the mucosa should always be biopsied if the diagnosis is uncertain [2, 5, 6, 8, 13].

## 4. Conclusion

As amalgam fillings still are ubiquitous and amalgam tattoos remain one of the most common causes of intraoral pigmentation, we consider amalgam tattoos to be an important differential diagnostic consideration, when assessing patients suspect for mucosal melanoma of the oral cavity. Information regarding previous prosthetic dental work should be included in the patient's medical history, and an X-ray showing metal deposits in the mucosa can safely rule out mucosal melanoma. But when in doubt, we recommend a diagnostic biopsy for histopathological examination.

## Figures and Tables

**Table 1:** Causes of intraoral pigmentation.

Endogenous	Exogenous
*Hereditary or congenital *	*Medicationand toxicity related *
Physiologic pigmentation (skin type 5-6)	Antimicrobial agents
Peutz-Jeghers syndrome	Antiarrhytchmic agents
Laugier-Hunziker syndrome	Oral contraceptives
	Cytostatics
*Systemic or infectious *	Smokers melanosis
Addison's disease	
Petechiae, varices, or thrombus	*Traumatic *
	Haematoma
*Neoplastic or melanin * *related *	Postinflammatory pigmentation
Naevi	Oral melanoacanthosis
Pigmented maculae	
Mucosal melanoma	*Other exogenous pigmentations *
Kaposi's sarcoma	Amalgam tattoos
Haemangioma	Accidental graphite tattoos
	Tribal tattoos
